# The Interplay Between the Gut Microbiota and Colorectal Cancer: A Review of the Literature

**DOI:** 10.3390/microorganisms13061410

**Published:** 2025-06-17

**Authors:** Marco Cintoni, Marta Palombaro, Eleonora Zoli, Giuseppe D’Agostino, Gabriele Pulcini, Elena Leonardi, Pauline Raoul, Emanuele Rinninella, Flavio De Maio, Esmeralda Capristo, Antonio Gasbarrini, Maria Cristina Mele

**Affiliations:** 1 UOC di Nutrizione Clinica, Dipartimento di Scienze Mediche e Chirurgiche, Fondazione Policlinico Universitario A. Gemelli IRCCS, Largo A. Gemelli 8, 00168 Rome, Italy; marta.palombaro@guest.policlinicogemelli.it (M.P.); eleonora.zoli@guest.policlinicogemelli.it (E.Z.); gabriele.pulcini@guest.policlinicogemelli.it (G.P.); elena.leonardi@guest.policlinicogemelli.it (E.L.); paulineceline.raoul@policlinicogemelli.it (P.R.); emanuele.rinninella@unicatt.it (E.R.); mariacristina.mele@unicatt.it (M.C.M.); 2 Centro di Ricerca e Formazione in Nutrizione Umana, Università Cattolica del Sacro Cuore, 00168 Rome, Italy; esmeralda.capristo@unicatt.it (E.C.); antonio.gasbarrini@unicatt.it (A.G.); 3 Azienda Ospedaliera di Cremona, 26100 Cremona, Italy; giuseppe.dagostino@asst-cremona.it; 4 Department of Laboratory and Hematological Sciences, Fondazione Policlinico Universitario A. Gemelli IRCCS, Largo A. Gemelli 8, 00168 Rome, Italy; flavio.demaio@policlinicogemelli.it; 5 UOS Medicina della Grande Obesità, Dipartimento di Scienze Mediche e Chirurgiche, Fondazione Policlinico Universitario A. Gemelli IRCCS, Largo A. Gemelli 8, 00168 Rome, Italy; 6 UOC Medicina Interna e Gastroenterologia, Dipartimento di Scienze Mediche e Chirurgiche, Fondazione Policlinico Universitario A. Gemelli IRCCS, Largo A. Gemelli 8, 00168 Rome, Italy

**Keywords:** gut microbiota, colorectal cancer, cancer treatment, radiotherapy, immunotherapy, chemotherapy

## Abstract

Lifestyle, diet, and genetics are established risk factors for developing colorectal cancer (CRC). In recent years, the role of the gut microbiota (GM) has been increasingly highlighted in several studies, suggesting an effect on both the disease’s pathogenesis and the efficacy and tolerability of treatments. We conducted a search on Medline, aiming to identify published studies exploring the role of the GM in the development and treatment of CRC. Dysbiosis, an imbalance in GM, is common in CRC patients and is associated with precancerous lesions, aggressive tumors, and varied therapy outcomes. Restoring GM balance can reduce treatment complications and may improve prognosis. The review details how GM influences CRC through metabolite production, inflammation modulation, and immune response alteration. Diet significantly impacts GM composition, with processed meats and high-fat diets increasing CRC risk, while fiber-rich diets are protective. The role of the GM in CRC treatments like surgery, chemotherapy, radiotherapy, and immunotherapy is also explored, noting its influence on complications, chemoresistance, and treatment efficacy. Future strategies involving GM modulation through diet, probiotics, and fecal microbiota transplantation (FMT) show promise for CRC prevention and treatment, warranting further research.

## 1. Introduction

Colorectal cancer (CRC) stands as one of the most prevalent and lethal malignancies globally, ranking as the fourth most common cancer [1]. Its pathogenesis, while not fully elucidated, is closely linked to the genetic instability of key mutated genes, triggered and modulated by a complex interplay of environmental and genetic risk factors. Among these, Western lifestyle habits, characterized by smoking, being overweight, excessive alcohol consumption, a diet rich in red and processed meats, low fiber and calcium intake, and sedentary behavior, emerge as significant determinants. However, the pivotal role of the gut microbiota (GM) in CRC pathogenesis has garnered increasing research attention in recent years [2].

The GM, a complex and dynamic ecosystem harboring over 100 trillion microorganisms, plays a fundamental role in human health, influencing immune, metabolic, structural, and neurological functions. From birth to adulthood, the human microbiota grows dynamically, responding to various internal and environmental influences. In healthy adults, the composition of the intestinal microbiota is generally stable throughout time [3]. The largest number of microorganisms found in the human body live in the large intestine, and its composition, dominated by the bacterial phyla *Firmicutes* and *Bacteroidetes*, representing over 90% of the GM, is modulated by environmental factors, including diet. It can undergo alterations, termed ‘dysbiosis’, which have been associated with various pathological conditions, including CRC [4].

The role of GM in colorectal carcinogenesis is consistently highlighted in numerous studies. Factors such as obesity, a high-fat diet, smoking, and frequent alcohol consumption all affect the Intestinal ecosystem and are thought to be related to colon carcinogenesis [5].

Research has highlighted the intricate mechanisms through which GM influences the development and progression of colorectal carcinogenesis. The gut microbiome, comprised of trillions of microorganisms, plays a pivotal role in various physiological processes, including digestion and immune regulation. However, recent studies have illuminated its potential role in the pathogenesis of CRC through several key mechanisms. One of the significant pathways by which the gut microbiome contributes to colorectal carcinogenesis is through the production of pro-carcinogenic metabolites. For instance, microbial-derived compounds such as secondary bile acids, hydrogen sulfide, and N-nitroso compounds have been shown to induce genotoxicity in colonic epithelial cells. These metabolites are capable of causing DNA damage and genomic instability, which are critical steps in the development of cancer. Furthermore, the gut microbiome can modulate chronic inflammation, a well-recognized risk factor for CRC. Dysbiosis, an imbalance in the microbial composition, can lead to persistent inflammation in the gut, which promotes an environment conducive to tumor development. The presence of certain pathogenic microbes, such as *Fusobacterium nucleatum* and *Bacteroides fragilis*, has been associated with increased inflammatory responses that may facilitate cancer progression. Additionally, the alteration of immune responses in the host is another area where the gut microbiome exerts influence. Certain microbial taxa can interact with the host’s immune system, leading to either an exacerbated inflammatory response or a state of immune tolerance. This dynamic can affect tumor surveillance by the immune system, thereby impacting the likelihood of tumor development and progression. Specific microbes have been heavily studied for their association with CRC. *F. nucleatum*, known for its role in periodontal disease, has been implicated in promoting tumor growth and metastasis. Similarly, specific strains of Escherichia coli that possess the polyketide synthase island have been identified as potential contributors to colorectal carcinogenesis due to their ability to produce genotoxic and pro-inflammatory substances. Beyond these direct effects, the interaction between the gut microbiome and the metabolome, particularly in polyamine metabolism, is emerging as a promising area of research. Polyamines are organic compounds that play essential roles in cell growth and differentiation, and alterations in their metabolism can have significant implications for cancer development. Understanding these interactions may pave the way for identifying novel diagnostic biomarkers as well as developing innovative therapeutic strategies aimed at modulating the gut microbiome to prevent or treat CRC. Overall, the evolving research on the gut microbiome and its multifaceted roles in colorectal carcinogenesis underscores the need for continued exploration in this field. As we unravel the complexities of these interactions, we may uncover new avenues for clinical intervention, contributing to improved outcomes for individuals at risk of developing CRC [6].

Diet-induced modifications to gut microbial composition and diversity are increasingly recognized to perturb the commensal-pathobiont balance, resulting in dysbiosis and a microenvironment conducive to cancer development [7]. Evidence indicates that augmenting dietary fiber intake may mitigate CRC risk, whereas high consumption of red and processed meat, saturated fats, and simple sugars negatively influences GM composition and diversity. Moreover, the GM mediates dietary effects on the body irrespective of cause and can enhance CRC risk by redirecting dietary impacts toward colonic neoplasia [7].

Understanding the role of the GM in CRC offers new perspectives for the prevention, early diagnosis, and treatment of this malignancy. The modulation of the GM through dietary interventions, probiotics, prebiotics, or fecal microbiota transplantation may represent a promising strategy to reduce CRC risk or improve the response to oncological treatments [8].

The GM composition undergoes modifications during the therapeutic pathway of CRC patients, from the preoperative phase (which includes bowel preparation with osmotic agents, preoperative antibiotic prophylaxis, etc.) to chemotherapy or radiotherapy, and these modifications can either improve or worsen the efficacy of the therapies [9].

This article aims to provide an updated overview of the scientific evidence regarding the role of the GM in CRC, analyzing the mechanisms through which the GM influences colorectal carcinogenesis and discussing the potential clinical implications of these findings. Herein, we comment on the latest findings on this topic.

## 2. Materials and Methods

This review was conducted on Medline from January 2019 to September 2024, aiming to identify the published studies exploring the role of GM in the development and treatment of CRC. The inclusion criteria for the studies were as follows: (i) observational, prospective, and retrospective studies, case-control studies, cohort studies, narrative reviews, systematic reviews, and meta-analyses; (ii) studies including information about the GM on CRC development or the treatment of patients; and (iii) studies written in English. All the studies that did not fall into the previous criteria were excluded from the review process. All graphical representations in this paper were hand-drawn by the authors with digital illustration software (Canva software, https://www.canva.com/en_in/, accessed online on 3 June 2025).

## 3. Gut Microbiota and Colorectal Cancer Development

The GM in healthy individuals consists of a predominance of Gram-negative *Bacteroides* and Gram-positive *Firmicutes*, while *Verrucomicrobia* and *Actinobacteria* represent a smaller portion. GM phyla differ in terms of type and number of genera. The *Firmicutes* phylum is made up of more than 200 different genera, including *Lactobacillus*, *Enterococcus*, *Bacillus*, *Clostridium*, and *Ruminicoccus*, while the *Actinobacteria* phylum consists mainly of the genus *Bifidobacterium*. The healthiness of the GM resides in its richness and heterogeneity. The decrease in commensal bacterial strains and, on the other hand, the increase in harmful bacterial species (opportunistic pro-inflammatory pathogens), alters intestinal permeability, leading to bacterial translocation, the activation of innate and adaptive immunity, and chronic inflammation [10].

Many studies have highlighted the presence of specific bacteria in CRC tissues and CRC patients’ fecal samples compared to healthy controls [11,12,13,14].

**Figure 1:**
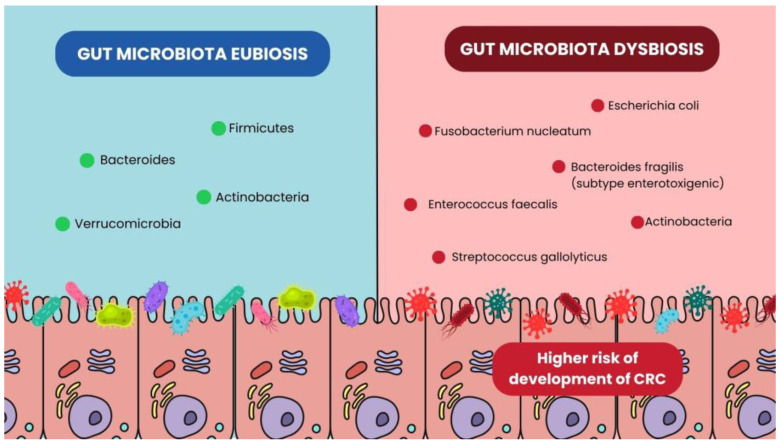
Bacterial differences in Gut Microbiota eubiosis and dysbiosis conditions.

Several species of CRC-associated bacteria have been functionally classified according to their mechanisms of action. A graphical summary is reported in Figure 1.

### 3.1. Genotoxin-Producing Bacteria

*Escherichia coli* seems to play an active role in the development of CRC. Ambrosi et al. showed that *E. coli* strains colonizing adenomatous polyps have a different phenotype as well as greater virulence than those present in the healthy mucosa [12]. Indeed, the notion that *Escherichia coli* could have carcinogenic properties was strengthened when a cytotoxic strain was discovered to harbor a genomic island—known as the pks island—encoding polyketide synthase (PKS) and non-ribosomal peptide synthase (NRPS) genes. This genomic island confers the ability to induce DNA double-strand breaks in mammalian cells. Further studies validated the carcinogenic potential of pks-positive *E. coli* (*pks⁺ E. coli*) by demonstrating an enrichment of the pks island in the colonic mucosa of patients with CRC and inflammatory bowel disease (IBD). Moreover, *pks⁺ E. coli* was shown to promote tumorigenesis in germ-free, IL-10-deficient mice, providing compelling in vivo evidence of its oncogenic role [15].

Moreover, Wilson et al. documented that colibactin, a genotoxin produced by particular strains of *E. coli*, such as *PKS+ E. coli*, can cause permanent DNA damage in vitro by alkylation of the DNA on adenine residues [16].

### 3.2. Inflammation-Associated Bacteria

Although *Bacteroides fragilis* is a commensal anaerobic bacterium, the subtype enterotoxigenic (ETBF) can promote colorectal carcinogenesis through the production of pro-inflammatory cytokines and stimulating the Wnt signaling pathway. Moreover, the expression of a *Bacteroides fragilis* toxin, a metalloprotease enzyme, can induce E-Cadherin cleavage, resulting in greater paracellular permeability and stimulation of B-catenin signaling, leading to cell proliferation [17]. Indeed, some authors proposed that the *ETBF* colonization of precancerous and cancerous lesions is a possible prognostic marker of CRC during screening [18].

*Fusobacterium nucleatum* is an oral symbiont and opportunistic pathogen that has been detected in intestinal cancers. It may play a role in carcinogenesis, as its occurrence appears to rise at various stages of CRC development. One of the mechanisms through which *Fusobacterium nucleatum* induces cell proliferation has been shown to depend on the bacterial adhesion molecule FadA. This protein facilitates the attachment to and invasion of epithelial cells by binding to the host cell-surface receptor E-cadherin. The interaction between FadA and E-cadherin leads to the activation of β-catenin signaling, which in turn promotes the transcription of genes involved in cell proliferation. Specifically, this activation results in increased expression of transcription factors and oncogenes such as c-Myc and Cyclin D1, contributing to tumor progression [19]. Using quantitative PCR techniques, it was found that CRC cases with high *Fusobacterium* presence (FB-high) were associated with specific molecular subtypes exhibiting potentially more aggressive tumor characteristics, including CpG island methylator phenotype (CIMP) positivity, microsatellite instability (MSI), and a high number of somatic mutations [20]. *Lactobacillus bulgaricus* has been found to reduce colitis-associated cancer by negatively regulating intestinal inflammation [21,22].

### 3.3. Other Potentially Pro-Oncogenic Bacteria

A further Gram-positive bacterial species closely associated with CRC is *Streptococcus* subspecies *gallolyticus* (*Sgg*). This microorganism possesses characteristics that allow it to colonize the tumor microenvironment and influence neoplasia progression. A distinctive characteristic of *Sgg* is the degradation of tannic acids (TAs), a dietary compound with anti-neoplastic properties, suggesting a potential impact on tumor growth modulation [23].

*Enterococcus faecalis* is a Gram-positive facultative anaerobic commensal bacterium, which, according to Wang X et al., could contribute to CRC starting processes in mouse colon epithelial cell models [24]. However, *Enterococcus faecalis* involvement in the occurrence and development of CRC is still debated; some studies point out a protective role, whereas others underline a pro-oncogenic activity [25].

Pro-oncogenic action has been linked to the generation of extracellular superoxide, which may cause DNA damage and genomic instability in the colonic epithelium, while the protective role correlates to certain species of *E. faecalis* that could be beneficial, as they could help maintain the integrity of the gut barrier and regulate the immune defense of the host.

This discrepancy likely reflects the variability of in vitro vs. in vivo systems, strain specificities, and the greater microbial context that *E. faecalis* typically inhabits. Moreover, host factors, including genetic background and immune status, could also affect the result of host–microbe interactions.

*Lactobacillus plantarum* produces indole-3-lactic acid, which has been shown to improve colorectal tumorigenesis by epigenetically regulating CD8+ T cell immunity [21].

## 4. Diet as a Risk Factor for CRC Development

The rising global incidence of CRC closely aligns with the adoption of the Western lifestyle [26,27]. A crucial link in this progression is the gut microbiota, which mediates chronic inflammation, a known driver in various cancer stages, including those seen in cardiometabolic disorders and inflammatory bowel disease [28].

Evidence consistently reveals significant differences in the fecal microbiome between CRC patients and healthy individuals. This CRC-associated dysbiosis mirrors that of IBD, featuring reduced microbial diversity, a decrease in beneficial anti-inflammatory bacteria, and an increase in opportunistic pathobionts [29]. A specific bacterial signature for CRC includes an enrichment of *Bacteroides fragilis*, *Enterococcus*, *Escherichia*/*Shigella*, *Klebsiella*, *Streptococcus*, and *PeptoStreptococcus*, while beneficial butyrate-producing bacteria like *Roseburia* and *Lachnospiraceae* are impoverished. Healthy individuals, conversely, show more *Bacteroides vulgatus* and *Bacteroides uniformis* [30]. Other analyses note CRC cases having enriched *Proteobacteria* and *Dorea* spp., but reduced *Bacteroidetes*, *Bacteroides* spp., and *Coprococcus* spp. [10].

Prominent pro-carcinogenic species, typically scarce in healthy individuals but linked to adenomas and CRC, include *Fusobacterium nucleatum*, *Bacteroides fragilis*, and *Parvimonas micra*. Their proliferation may promote dysplasia and mucosal invasion. These bacteria can induce a pro-inflammatory microenvironment or exert direct oncogenic actions by producing toxins or reactive oxygen species that damage DNA. For instance, *Fusobacterium* spp. are consistently associated with CRC, potentially through inflammatory mechanisms [29].

Conversely, the gut microbiota also modulates the bioavailability and properties of various dietary compounds. It transforms phenolic compounds into smaller, more bioavailable metabolites and is crucial for modulating pro-anthocyanidins bioavailability. These pro-anthocyanidins can exert local beneficial actions on colonic epithelial cells, offering protection against inflammation-mediated diseases like CRC [31].

These findings collectively underscore that dietary factors profoundly influence gut microbiota composition, creating imbalances that contribute significantly to CRC etiology. Understanding these intricate diet–microbiota interactions is vital for developing microbiota-manipulation strategies for CRC prevention and identifying at-risk individuals [32].

**Figure 2:**
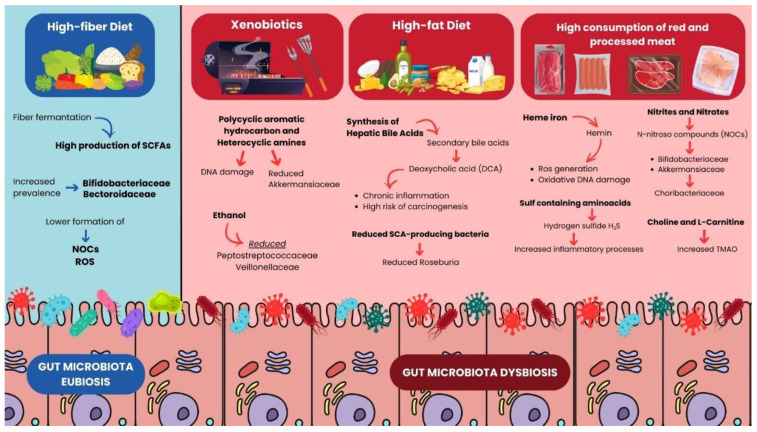
Impact of dietary patterns and xenobiotics on gut microbiota and CRC risk.

Figure 2 graphically illustrates a comprehensive resume.

### 4.1. Red and Processed Meat

Red and processed meat intake is one of the most consistently reported dietary factors linked to CRC. Red meat is characterized by an elevated content of Heme Iron, which can easily catalyze the generation of reactive oxygen species (ROS) in colon epithelial cells. Seiwert et al. (2020) evidenced that hemin—the oxidized form of heme—may induce ROS generation and oxidative DNA damage, thereby increasing the likelihood of mutagenesis in normal colonocytes [33]. Such effects contribute to a pro-oxidative colonic setting that promotes genetic instability and tumor initiation. The overproduction of ROS can disrupt cellular defense mechanisms, including the induction of heme oxygenase-1 (HO-1), resulting in DNA filament breaks and facilitating carcinogenic transformation [33,34]. In processed meats, the incorporation of nitrites and nitrates is a significant concern. These preservatives interact with amines during processing or digestion to form N-nitroso compounds (NOCs), which are powerful mutagens [6]. Rizzolo-Brime et al. (2024) reported that a high intake of nitrosyl-heme compounds from processed meats is associated with a shift in the GM, including a decrease in beneficial bacteria such as *Bifidobacteriaceae* [35]. Furthermore, the metabolism of sulfur-containing amino acids in red meat by gut bacteria produces hydrogen sulfide (H_2_S), a compound shown to promote mucosal damage by triggering inflammatory processes in the colon [36,37]. Overall, these results tend to suggest that red and processed meats impact CRC risk through not only chemical damage directly but also through modulating gut microbial populations. All these molecular mechanisms together may contribute to the creation of a microenvironment favoring colorectal carcinogenesis [38]. A pilot study reported that diets containing high levels of red meat are correlated with decreased *Bacteroidaceae* and *Akkermansiaceae*, while enhancing the growth of potentially pro-inflammatory taxa such as *Choriobacteriaceae* [39]. In addition, increased serum levels of trimethylamine N-oxide (TMAO), a microbiota-derived metabolite from choline and L-carnitine abounding in red meat, have been associated with a higher risk of CRC, especially in the distal colon [40].

### 4.2. Fiber-Rich Diet

On the other hand, epidemiological data support the beneficial role of high-fiber and vegetable-rich nutritional plans, which seem to provide a protective effect against CRC [41]. Dietary fibers withstand digestion processes in the upper gastrointestinal tract and are fermented by the GM in the colon. These processes permit the production of short-chain fatty acids (SCFAs) such as butyrate [42]. Butyrate is the main energy source for colonocytes and exhibits anti-inflammatory and anti-neoplastic properties by promoting apoptosis in transformed cells and maintaining mucosal barrier integrity [28,43].

Controlled studies of dietary intervention have shown evidence of the preventive role of fiber. O’Keefe et al. showed that rural Africans who consume diets with high fiber and low meat content have significantly reduced levels of CRC and a distinct gut microbial profile marked by an increased prevalence of beneficial taxa such as *Bacteroidaceae* and *Bifidobacteriaceae* [44]. Moreover, studies indicate that increasing fiber intake in high-risk populations enhances SCFAs production and shifts the microbiota toward a less inflammatory and more resilient composition to genotoxic insults [45]. Anti-carcinogenic effects of fiber-rich diets extend to the possible ability to dilute and modulate the exposure of the colon to potentially harmful dietary xenobiotics, thereby reducing the formation of NOCs and ROS, as suggested especially by cellular and animal models [45,46].

### 4.3. High-Fat Diet

High-fat nutritional patterns are also linked to increased risk of CRC, mainly through their effects on bile acid metabolism and the GM. Highly saturated fat diets boost the synthesis of hepatic bile acid. The excess bile acids reach the colon and are metabolized by intestinal bacteria into secondary bile acids like deoxycholic acid (DCA) [47,48]. DCA is known to induce chronic inflammation and direct epithelial injury, conditions conducive to tumorigenesis [49,50].

Animal studies have demonstrated that high-fat diets alter the bile acid pool and cause significant shifts in the GM. For instance, high-fat feeding in mice induces dysbiosis and reduces levels of SCFA-producing bacteria such as *Roseburia* [46,47]. These microbial shifts exacerbate the inflammatory environment within the colon and impair mucosal barrier function, thereby facilitating CRC development in murine models [51,52]. Retrospective cohort studies support these findings, linking high-fat diets to a higher incidence of colorectal neoplasia and to the activation of the MCP-1/CCR2 axis in CRC patients [51]. A cross-sectional study of Alaskan patients demonstrated that high-fat diets were associated with a tumor-promoting colonic milieu, as indicated by high rates of adenomatous polyps—a finding that may be mediated by low dietary fiber intake [53]. Comparable observations were reported in South Africa, where urban adults who consumed high-fat, low-fiber diets exhibited decreased fecal microbiome diversity, a shift from *Prevotella* to taxa linked to bile acid metabolism, and high levels of deoxycholic acid [54]. High-fat diets are also commonly linked to both obesity and metabolic syndrome, conditions that additionally increase the risk of CRC through chronic inflammation and microbial dysbiosis [55,56].

### 4.4. Xenobiotics

The interactions involving diet and the GM are complicated further by xenobiotics generated during food preparation and cooking. In situations when meat is cooked at high temperatures, compounds such as polycyclic aromatic hydrocarbons (PAHs) and heterocyclic amines (HAs), including PhIP and MeIQx, are produced. These molecules can directly lead to DNA damage [35]. Ruiz-Saavedra et al. found that high intake of these xenobiotics is associated with specific shifts in the GM; for example, diets with elevated PAH levels correlate with reduced *Bacteroidaceae* and increased *Coriobacteriaceae*, while high HA intake is linked to a decreased abundance of *Akkermansiaceae* [39]. Additionally, alcohol consumption, common in Western diets, further disrupts the microbial balance. In subjects with intestinal polyps, ethanol intake above 12 g per day is associated with an increased abundance of *Peptostreptococcaceae* and a decreased presence of *Veillonellaceae*, thereby promoting the metabolic activation of pro-carcinogens and contributing to a higher CRC risk [39,40].

## 5. Gut Microbiota and Colorectal Cancer Treatments

Emerging evidence indicates that the GM plays a vital role throughout the cancer journey. The GM undergoes significant changes during various cancer treatments, which can influence therapeutic outcomes in important ways. Understanding the complex relationship between the gut microbiome and cancer treatment is crucial for developing personalized therapeutic strategies. By leveraging insights from the GM, healthcare providers may be able to optimize treatment plans, mitigate side effects, and ultimately improve patient outcomes. As more significant data continues to emerge, the potential for the gut microbiome to serve as a therapeutic target or biomarker in oncology appears increasingly promising [9].

### 5.1. Surgery

The GM can play a crucial role in CRC surgery. The preoperative use of mechanical bowel preparation (MBP) is a common practice in colorectal surgery, even if its use is discouraged by the Enhanced Recovery After Surgery (ERAS) Guidelines [57]. MBP effectively reduces the solidity of fecal matter, thereby facilitating the surgeon’s ability to directly palpate, identify, and manipulate small CRC with greater ease, but as demonstrated by the current literature, its use does not affect the prevention of postoperative complications such as leakage of anastomosis [58].

The use of MBP determines heavy alterations of the luminal and mucosal microbiota, mostly immediately after MBP, with a complete restoration of basal GM only after 14 days. In particular, there is a decrease in *Bifidobacteria*, *Clostridium coccoides*, *Clostridium leptum*, *Enterobacteriaceae*, and *Lactobacillus* populations after surgery [59]. These alterations of microbiota and the decrease in SCFAs levels could lead to an increase in intestinal permeability with consequent bacterial translocation and proliferation of pathogenic species such as *Escherichia coli* and *Staphylococcus* [60].

As regards antibiotic prophylaxis, many studies have shown that the use of antibiotics in CRC surgery can lead to profound GM changes, reducing the heterogeneity of the intestinal microbiota from the first day of administration and obtaining a full GM recovery only after 60 days. Moreover, several studies showed that the intravenous administration of β-lactams (cefazolin, ampicillin, sulbactam, etc.) led to an increase in *Firmicutes* and a decrease in *Bacteroidetes* at the end of treatment [61].

GM composition can influence postoperative complications; in particular, infections seem to be associated with a distinct dysbiosis profile [62].

It is a fact that the GM plays a key role in the development of anastomotic leak (AL), one of the most frequent complications of CRC surgery. In fact, several bacteria have adhesive properties and can colonize the anastomotic site, detrimentally affecting the restitution of the epithelial barrier. Specific bacteria with the ability to produce collagenolytic enzymes and activate matrix metalloproteinase 9 (MMP-9) are involved in the development of AL. In particular, *Enterococcus faecalis* has a strong ability to degrade collagen directly and through the active conversion of matrix proteins, so much so that its presence in the drainage after surgery could be useful for detecting early AL [63]. Moreover, *Enterococcus faecalis* can exploit the human fibrinolytic protease plasminogen (PLG), leading to supraphysiological collagen degradation and a consequent high risk of surgery-related complications such as anastomotic leak and perforation [64]. The use of the PLG inhibitor tranexamic acid (TXA) is associated with a significant reduction in clinically relevant complications in murine models [64]. Palmisano et al. compared GM composition among patients who developed AL and those who had an eventful recovery after CRC surgery. By analyzing the GM from stool samples collected before surgery and after neoadjuvant therapy, they found a prevalence of *Acinetobacter lwoffii*, *Acinetobacter johnsonii*, and *Hafnia alve* in patients who experienced AL. On the contrary, *Barnesiella intestinihominis*, well represented in the group without complications, was absent in the group with AL [65].

Postoperative ileus (POI), the absence of intestinal motility following a surgical procedure, is another common complication after CRC surgery. Very few studies in the literature have looked for a link between POI and the GM, even if some evidence is present [66,67,68]. When comparing the GM of CRC patients with and without ileus, the *Firmicutes*/*Bacteroidetes* ratio and microbial dysbiosis index (MDI) showed greater dysbiosis among ileus patients. Moreover, *Faecalibacterium* appears to be significantly reduced in patients with POI, thus representing a potential biomarker for predicting such complications [67]. Recent research on the guinea pig model assessed the benefit of GM modulation by probiotics in the treatment and prevention of POI [68].

### 5.2. Chemotherapy

There is a two-way interaction between the GM and chemotherapy (CHT). Indeed, after a CHT treatment, a decrease in the variability and abundance in the GM is found, as well as dysbiosis. On the other hand, GM composition may influence chemotherapy efficacy and tolerance.

In recent years, several studies have demonstrated the role of the GM in the response to a range of chemotherapies in CRC as well as other tumors.

CRC can be treated using different cytotoxic chemotherapeutic agents, but there are three main chemotherapy regimens for advanced CRC treatment: XELOX (Capecitabine + Oxaliplatin), FOLFIRI (Folinic Acid + 5-Fluorouracil (5-FU) + Irinotecam), and FOLFOX (Folinic Acid + 5-FU + Oxaliplatin) [69]. There is sufficient evidence to say that *Fusobacterium nucleatum* is involved in the mechanisms of chemoresistance [70,71]. In a recent study, resistance to 5-FU was found to be linked, both in vitro and in vivo, with *Fusobacterium nucleatum* infection. In fact, it seems to reduce the chemosensitivity of CRC cells through the upregulation of BIRC3, a gene that encodes for a baculoviral protein that interferes with the caspase cascade and inhibits cellular apoptosis [72]. In contrast with these findings, a retrospective analysis conducted on tissue from surgically collected specimens of 593 CRC patients who had undergone curative surgery and subsequent adjuvant chemotherapy (either FOLFOX or XELOX) did not find any significant differences in survival between the *F. nucleatum*-high and -low/negative groups [73].

Summarizing the above, it has been reported that in vitro and in vivo *Fusobacterium nucleatum* is able to enhance 5-FU-resistance by blocking apoptosis. Nevertheless, meta-analysis using clinical data from patients with CRC showed no definite effect on clinical efficacy, suggesting that there was a discrepancy between preclinical and human evidence.

In a randomized, single-blind, placebo-controlled prospective study, the administration of probiotic tablets containing a combination of *Bifidobacterium infantis*, *Lactobacillus acidophilus*, *Enterococcus faecalis*, and *Bacillus cereus* from the third postoperative day until the end of the first chemotherapy course (XELOX regimen) demonstrated efficacy in reducing gastrointestinal complications without altering the efficacy of chemotherapy [74].

As for neoadjuvant treatment, a prospective longitudinal study on 84 patients with locally advanced rectal cancer confirmed that the overexpression of *Fusobacterium* was associated with poor response to chemoradiotherapy (conventional radiotherapy + concurrent capecitabine-based chemotherapy). On the other hand, “beneficial microbiota”, made of butyrate-producing microbes, such as *Roseburia*, *Dorea*, and *Anaerostipes*, was overrepresented in responders. *Streptococcus*, which significantly increases after neoadjuvant treatment, was suggested as a potential biomarker for predicting chemoradiotherapy response [75].

During CHT, many adverse events can affect patients’ quality of life as well as chemotherapy efficacy, causing delays or treatment interruptions. Gastrointestinal mucositis and chemotherapy-induced diarrhea (CID) are common side effects during antitumor treatment with cytotoxic drugs in CRC.

Gastrointestinal mucositis is a fearful adverse event that can severely affect drug tolerance during 5-FU treatment. Multiple mechanisms contribute to the complicated pathogenesis of 5-FU-induced intestinal mucositis (FUIIM), including direct toxicity, oxidative stress, abnormal inflammation, and changes in the balance of bowel microbial flora. Recent research indicates that regulating the GM is a potential target for treating FUIIM [76,77].

Interestingly, differences in microbial flora occur between patients who experienced complications during CHT and those who did not. From the analysis of stool samples of 17 CRC patients after 2 weeks of treatment with the adjuvant CapeOx regimen, GM community richness and diversity appear to be lower in patients who experienced CID compared with the control group. Notably, a high concentration of *Klebsiella* pneumoniae was noticed in patients with CID [78]. Probiotics administration can protect against chemotherapy-induced dysbiosis, promote SCFAs production, and be effective in limiting gastrointestinal complications [74].

### 5.3. Immunotherapy

The host immune system represents a powerful tool in the fight against cancer. Both cell-mediated and adaptive immune responses can lead to tumor regression.

In recent years, immunotherapy has achieved a leading role in the treatment of a variety of neoplasms with extended response and increased survival rate. In any case, the application of immunotherapy in CRC patients is limited. It is approved only for CRC with mismatch repair-deficient (dMMR) or microsatellite instability-high (MSH) [79].

An important class of immunotherapy drugs is the immune checkpoint inhibitors (ICIs). They act against cancer by breaking the mechanism that neoplastic cells use to escape immune surveillance. ICIs have two major targets: the cytotoxic T-lymphocyte-associated protein 4 (CTLA-4) and the programmed death receptor and its ligand 1 (PD1/PD-L1). Anti-PD1 pembrolizumab is a promising monotherapy first-line treatment for metastatic CRC with dMMR or MSH [80].

The GM modulates the immune system and can represent a strategy to ameliorate the therapeutic response and toxicity of cancer immunotherapy. To date, studies have focused mainly on melanoma patients. Here, they evidence a significant difference in GM composition between responders and non-responders, with enhanced GM diversity in responders [81]. The use of antibiotics inhibits the benefits of ICIs, and fecal microbiota transplantation (FMT) in responding patients in germ-free mice ameliorated the antitumor effects of anti-PD-1 blockade [81,82]. In non-small cell lung cancer and renal cell carcinoma, the abundance of *Akkermansia muciniphila* promotes the efficacy of ICIs [82,83]. Moreover, the metagenomics of patients’ stool samples revealed that specific metabolites, including SCFAs such as butyrate, can be considered as biomarkers of responsiveness [83,84]

Limited data is available on CRC. Recently, a study on CRC tumor-bearing mice also suggests how the gut microbiome plays a key role in the treatment of this type of tumor using the PD-1 antibody. The authors found that treatment with different antibiotics affects immunotherapy response [85].

In vivo evidence suggests that intervention with *Lactobacillus acidophilus* supports immune response and enhances the efficacy of anti-CTA-4 immunotherapy through the induction of effector memory T cells and CD8+ T-cells [86].

Recently, an analysis of fecal samples from patients with RAS wild-type metastatic colorectal cancer (mCRC) undergoing combination treatment with cetuximab and avelumab has highlighted that the presence of butyrate-producing bacterial species is associated with a better response to treatment and a longer progression-free survival (PFS), suggesting that the presence of these species may represent an interesting biomarker in the context of treatment response [87].

Colibactin-producing *E. coli (CoPEC)* are frequently detected in CRC and exhibit pro-carcinogenic activity. Lopèz A et al. found that the colonization of CRC patients by *CoPEC* is associated with a decrease in tumor-infiltrating T lymphocytes (CD3+ T cells). Similarly, in mice, chronic *CoPEC* infection decreases CD3+ and CD8+ T cells and reduces the efficacy of anti-mouse PD-1 immunotherapy. These findings suggest that *CoPEC* could promote a pro-carcinogenic immune environment by impairing the antitumor T-cell response, leading to tumoral resistance to immunotherapy [88].

### 5.4. Radiotherapy

In localized rectal cancer, neoadjuvant radiotherapy (RT) or chemoradiotherapy (CRT) is frequently used to improve local control and survival. The use in colon neoplasm is mostly confined to metastatic disease [89].

The GM can influence both the toxicity and effectiveness of RT. Radiations cause alterations in GM composition with evidence of dysbiosis. Exposure to ionizing radiation increases inflammation and membrane permeability so that bacterial translocation is promoted. This can lead to radiation-induced gastrointestinal mucositis [90].

Proctitis is a common side effect during radiotherapy for rectal cancer. In mice treated with localized internal rectal radiation, a significant change in bacterial phyla was noticed when compared with controls. Notably, six genera showed increased abundance in mice with radiation proctitis, including *Akkermansia*, *Bacteroides*, *ParaBacteroides*, *Sutterella*, *Turicibacter*, and an unclassified genus [91].

Altered microbiota induced mucosal TNFα and IL-1β secretion, which contributed to inflammation and toxicity. Treatment with IL-1 receptor antagonist reduces intestinal radiation-induced damage [91].

A link between an altered GM at the end of chemo and radiotherapy and fatigue was recently found through 16S rRNA gene sequencing of stool samples from 50 rectal cancer patients. Differentially abundant microbial taxa were identified, with enriched *Eubacterium*, *Streptococcus*, *Adlercreutzia*, and *Actinomyces*, as well as abundances of the microbial sucrose degradation pathway in fatigued patients [92].

## 6. Future Perspectives

### 6.1. Pre- and Probiotics

Modulation of the GM represents a strategy of significant impact in the prevention and treatment of CRC, but it currently poses a real challenge for physicians. Only a few studies have explored the effective role of administering diet, probiotics, and prebiotics before or during CRC therapies. Probiotics have anti-inflammatory and antiproliferative properties; thus, they may exert direct anticancer activity [93]. Furthermore, emerging evidence indicates that probiotics are capable of biotransforming chemical compounds and anticancer agents, influencing drug bioavailability and therapeutic outcomes [94]. Nowadays, probiotics are primarily utilized in the management of cancer treatment-related diarrhea; however, their application, as well as the use of prebiotic and probiotic-derived bioactive compounds, presents potential for increased therapeutic applications [74]. Recent research has shown that combining immunotherapy with probiotics in patients with mCRC is effective in improving symptomatology and overall quality of life, underscoring the need for further investigation [95].

A therapeutic approach targeting the microbiota in CRC treatment could derive from a personalized diet, given its impact on gut microbiome composition. Extensive epidemiological investigations have consistently shown a protective association between dietary habits and CRC incidence. A high intake of certain food categories is strongly implicated in the etiology of CRC [27]. Nevertheless, the current body of literature provides limited evidence regarding the impact of diet during oncological therapies and its potential to modulate treatment tolerance. Further research on this topic will contribute to refining dietary recommendations in this specific context.

### 6.2. Fecal Microbiota Transplantation

Fecal microbiota transplantation (FMT), widely used for the treatment of *Clostridium difficile* infection, shows promise as a novel strategy in other gastrointestinal disorders and in the prevention and treatment of CRC [96]. Moreover, emerging evidence in recent years indicated that FMT is highly effective in enhancing the response to immunotherapy in melanoma patients [97]. Although research on FMT in CRC is limited, pilot studies suggest it could improve patient outcomes, showing a demonstrated safety profile [98,99]. An exciting application field of microbiota transplantation is the treatment of radiation enteritis, a frequent complication of radiotherapy in pelvic cancers. The GM, severely damaged by radiation, can be effectively restored through this procedure, leading to a resolution of the patient’s symptoms [100].

## 7. Conclusions

Our review highlights the significant involvement of the GM in both the development and prognosis of patients with CRC. Specific bacterial strains (e.g., *E. coli*, *Fusobacterium nucleatum*, and *Enterococcus faecalis*) have been identified as potential markers for precancerous lesions, more aggressive disease, and differential responses to treatments. A summary of the principal results is given in Table 1.

Oncological treatments induce a significant perturbation of the GM, leading to an increased incidence of complications. The administration of prebiotics, probiotics, and postbiotics has the potential to restore the intestinal flora and thereby mitigate treatment-related side effects, with particular emphasis on mucositis and diarrhea. Furthermore, probiotics and their metabolites can directly influence the response to therapies by altering bioavailability. In particular, given its ability to modulate the immune system, the GM offers great potential in association with immunotherapy.

Interventions designed to address dysbiosis (dietary adjustments, FMT, probiotics administration, etc.) are essential in the prevention of CRC and clearly represent a valid strategy in conjunction with conventional therapies to support oncological patients.

Further clinical trials are still warranted on this topic to validate its promising clinical efficacy and to standardize its application.

## Figures and Tables

**Table 1:** Summary of treatment options for colorectal cancer and their impact on the gut microbiota.

Treatment Area	Specific Modality	Advantages/Positive Impacts	Disadvantages/Complications
Surgery	Mechanical Bowel Preparation	It facilitates the detection and handling of small CRCs by directed palpation by the surgeon.	It does not affect the prevention of postsurgical complications, such as anastomotic leaks. Severe alterations of the luminal and mucosal microbiota (e.g., reduction of *Bifidobacteria*, *Clostridium coccoides*, *Lactobacillus*), with total re-establishment only after 14 days. This may result in a greater intestinal permeability, translocation of bacteria, and proliferation of pathogenic species.
	Antibiotic Prophylaxis	-	Major GM changes reduced heterogeneity, with complete restoration only after 60 days. Intravenously administered β-lactams resulted in an overall increase in *Firmicutes* and a reduction in *Bacteroidetes*.
	General Colorectal Surgery	-	Postoperative complications, such as infections, are accompanied by a discrete dysbiosis pattern. Anastomotic leaks are affected by bacteria (e.g., *Enterococcus faecalis*, *Acinetobacter lwoffii*) that either colonize the anastomotic site, influence the epithelial barrier, biodegrade collagen, or activate MMP9. Postoperative ileus is also associated with increased dysbiosis and decreased *Faecalibacterium*
Chemotherapy (CHT)	Cytotoxic regimens		Chemoresistance related to specific bacteria (e.g., *Fusobacterium nucleatum* regulating BIRC3). Adverse reactions such as gastrointestinal mucositis and chemotherapy-induced diarrhea. Reduced GM diversity, dysbiosis and high *Klebsiella.*
	Neoadjuvant Chemoradiotherapy	The ‘beneficial microbiota’ (e.g., *Roseburia*, *Dorea*) is over-represented in responders. *Streptococcus* has been proposed as a potential biomarker to predict responsiveness.	*Fusobacterium* overexpression is linked to scarce responsiveness.
	Probiotics with CHT	It may decrease gastrointestinal side effects while not affecting the effectiveness of chemotherapy. It protects from chemotherapy-induced dysbiosis and supports the generation of short-chain fatty acids (SCFAs). It mitigates the treatment-related adverse reactions, in particular, mucositis and diarrhea.	There is limited research exploring the beneficial role before/during CRC treatments.
Immunotherapy	Immune Checkpoint Inhibitors	The GM may improve treatment response and toxicity. Greater diversity of the GM in responders (melanoma). The abundance of *Akkermansia muciniphila* enhances the efficacy of ICIs (other cancer types). The metabolites, such as SCFA (e.g., butyrate), may be biomarkers of responsiveness. The occurrence of butyrate-producing bacteria is linked to a better response to cetuximab + avelumab in mCRC.	Very limited application to CRC (only approved for CRC dMMR or MSH). Antibiotics inhibit the beneficial effects of ICIs. Colonization by colibactin-producing *E. coli* (CoPEC) can decrease effectiveness by compromising anti-cancer T-cell sensitivity.
	*Lactobacillus acidophilus* with ICIs	It sustains the immune system response and boosts anti-CTLA-4 effectivity (mouse model).	
	Probiotics with Immunotherapy	Successful association in improving symptom burden and quality of life in metastatic CRC. The GM holds major potential through immune modulation.	Additional research is required.
Radiotherapy (RT)	Localized RT or CRT (for rectal cancer)	Enhances local management and overall survival in localized rectal carcinoma.	Altered GM composition; dysbiosis. Elevated inflammation, increased membrane permeability, and enhanced bacterial translocation. Radiation-induced gastrointestinal mucositis. Proctitis linked to alterations in bacterial phyla (e.g., increase in *Akkermansia*, *Bacteroides*) and the production of pro-inflammatory cytokines. Fatigue tied to GM alterations.
Gut Microbiota (GM) Modulation (Future Perspectives)	Dietary Interventions	Customized nutrition has an influence on GM composition. A protective influence between dietary patterns and incidence of CRC. Modulation potential of treatment tolerance. Crucial in preventing CRC.	Scarce available data on the impact during cancer therapies. Represents a major challenge for physicians.
	Probiotics/Prebiotics	Anti-inflammatory and anti-proliferative effects, direct anti-tumor action, and treat treatment-related diarrhea (probiotics). Repair intestinal flora, attenuate adverse effects (prebiotics, probiotics).	Only a few studies have investigated the true role before/during treatment for CRC. It represents a major challenge for clinicians. Additional studies are needed.
	Fecal Microbiota Transplantation (FMT)	It holds great potential for the prevention and treatment of CRC. It improves responsiveness to immunotherapy (melanoma). Pilot studies safely suggest an improvement in CRC outcomes. Affective for radiation enteritis, restoring the GM. Key in CRC prevention and support strategy.	Research on CRC is scarce. It represents a major challenge for clinicians. Additional research is needed.

## Data Availability

No new data were created or analyzed in this study. Data sharing is not applicable to this article.
